# *Basonuclin-2* Requirements for Zebrafish Adult Pigment Pattern Development and Female Fertility

**DOI:** 10.1371/journal.pgen.1000744

**Published:** 2009-11-26

**Authors:** Michael R. Lang, Larissa B. Patterson, Tiffany N. Gordon, Stephen L. Johnson, David M. Parichy

**Affiliations:** 1 Department of Biology, University of Washington, Seattle, Washington, United States of America; 2 Department of Genetics, Washington University School of Medicine, St. Louis, Missouri, United States of America; Stanford University School of Medicine, United States of America

## Abstract

Relatively little is known about the generation of adult form. One complex adult trait that is particularly amenable to genetic and experimental analysis is the zebrafish pigment pattern, which undergoes extensive remodeling during post-embryonic development to form adult stripes. These stripes result from the arrangement of three classes of neural crest-derived pigment cells, or chromatophores: melanophores, xanthophores, and iridophores. Here, we analyze the zebrafish *bonaparte* mutant, which has a normal early pigment pattern but exhibits a severe disruption to the adult stripe pattern. We show that the *bonaparte* mutant phenotype arises from mutations in *basonuclin-2* (*bnc2*), encoding a highly conserved, nuclear-localized zinc finger protein of unknown function. We show that *bnc2* acts non-autonomously to the melanophore lineage and is expressed by hypodermal cells adjacent to chromatophores during adult pigment pattern formation. In *bonaparte* (*bnc2*) mutants, all three types of chromatophores differentiate but then are lost by extrusion through the skin. We further show that while *bnc2* promotes the development of two genetically distinct populations of melanophores in the body stripes, chromatophores of the fins and scales remain unaffected in *bonaparte* mutants, though a requirement of fin chromatophores for *bnc2* is revealed in the absence of *kit* and *colony stimulating factor-1 receptor* activity. Finally, we find that *bonaparte* (*bnc2*) mutants exhibit dysmorphic ovaries correlating with infertility and *bnc2* is expressed in somatic ovarian cells, whereas the related gene, *bnc1*, is expressed within oocytes; and we find that both *bnc2* and *bnc1* are expressed abundantly within the central nervous system. These findings identify *bnc2* as an important mediator of adult pigment pattern formation and identify *bonaparte* mutants as an animal model for dissecting *bnc2* functions.

## Author Summary

The pigment patterns of animals are some of the most distinctive traits and serve as useful models for understanding the development of adult form more generally. In zebrafish, horizontal stripes result from the arrangements of several classes of pigment cells. Here, we used a mutational approach to identify a critical new gene required for stripe development, *basonuclin-*2, which encodes a highly conserved zinc finger protein that localizes to the nucleus. In contrast to other genes known in this species so far, which act within the pigment cells during pattern formation, we show that *basonuclin-2* is expressed and functions in the extracellular environment in which the pigment cells reside. Without *basonuclin-2*, pigment cells die and, literally, fall off the fish. However, the effects of *basonuclin-2* also extend beyond pigmentation, as female mutants have ovarian defects and are infertile, and the gene is expressed widely in the central nervous system, hinting at functions there as well. Our study thus reveals a critical component of the “canvas” on which these stunning pigment patterns are painted, and provides a new model for dissecting *basonuclin-2* roles in the development of adult form and function.

## Introduction

The mechanisms underlying the generation of adult form remain largely unknown, despite progress towards understanding the genes and cell behaviors responsible for morphogenesis in embryos and some organ systems. A useful system for studying how adult phenotypes are generated is the skin pigment pattern [1]–[3]. These patterns are among the most prominent features of many organisms and serve functions including camouflage, warning coloration, and the facilitation of social interactions ranging from species recognition to mate choice [4]–[6]. Besides their ecological and evolutionary relevance, pigment patterns also are especially useful and intriguing because of their diversity, which occurs even between closely related species. Adding to this diversity is pigment pattern variation within species that can arise stochastically, but also through deterministic changes at particular phases of the life cycle.

Vertebrate pigment cells in the skin are derived from embryonic neural crest cells [7],[8]. Yet, there is now considerable evidence that some adult pigment cells arise not directly from neural crest cells, but from post-embryonic, neural crest-derived stem cells (NCSCs)[9]–[13]. Such stem cells are self-renewing and can be pluripotent. Thus, vertebrate pigment patterns also serve as a model for understanding the mechanisms of stem cell establishment, maintenance, and recruitment to form particular aspects of adult phenotypes, either during normal development and homeostasis, or during repair and regeneration.

More than a century of studying pigment pattern mutants has allowed the identification of numerous loci required for pigment cell development and pattern formation [14]. Many of these mutants have overt phenotypes limited to the pigment cells themselves, often reflecting defects in pigment synthesis. Nevertheless, some mutants exhibit pleiotropic defects in other neural crest derivatives or other organ systems. Among the most famous of these are mammalian mutants for the kit receptor tyrosine kinase and its ligand, Steel factor [15]–[17]. These have defects not only in the development of neural crest (or NCSC)-derived pigment cells, but also in gametogenesis and hematopoiesis, reflecting failures in three distinct stem cell systems.

More recently, the zebrafish *Danio rerio* has emerged as a model system for studies of pigment pattern formation and stem cell biology. Unlike endothermic vertebrates that have a single neural crest-derived pigment cell—the melanocyte—zebrafish and other ectothermic vertebrates exhibit several classes of pigment cells, collectively referred to as chromatophores [1],[3],[18],[19]. These include black melanophores that contain melanin and are the ectotherm equivalent of melanocytes, as well as yellow or orange xanthophores that contain pteridines and carotenoids, and iridescent iridophores that contain purine-rich reflecting platelets. The arrangement of these cells generates the adult pigment pattern, consisting in zebrafish of horizontal dark stripes of melanophores and iridophores and light “interstripes” of xanthophores and iridophores [20],[21]. Several lines of evidence indicate the stripes form in part due to interactions between melanophores and xanthophores [22],[23] and the mechanics of stripe development are consistent with the action of a Turing mechanism [24],[25]. What other factors are required for adult pigment pattern formation remain largely unknown.

While numerous zebrafish pigment pattern mutants have now been described, and some of the corresponding genes identified, the majority of these mutants exhibit few if any discernible pleiotropic defects in other organ systems. For example, mutations in the *leopard* (*cx41.8*) and *jaguar* (*kir7.1*) genes seem to overtly affect chromatophores but not other tissues [26],[27]. Moreover, some zebrafish pigment pattern mutants display less severe defects than the corresponding mouse mutants. For example, null alleles of *sparse* (*kit*) in zebrafish or a closely related species retain substantial numbers of melanophores, whereas null alleles of *Kit* in mouse completely lack melanocytes [17],[28]. Likewise, *rose* (*endothelin receptor b1*, *ednrb1*) mutants in zebrafish retain numerous melanophores without other overt defects, whereas the corresponding *Ednrb* mutant of mouse lacks all melanocytes and develops aganglionic megacolon [29],[30]. Finally, zebrafish *nacre* (*microphthalmia-a*, *mitfa*) mutants lack nearly all melanophores, but are otherwise normal, in contrast to mouse mutants that completely lack melanocytes, exhibit reduced eye size, and have defects in mast cell and osteoclast development [31],[32]. These differences in phenotypic severity might be a consequence of an additional round of whole genome duplication in teleosts and differences in the partitioning of functions amongst homologous loci in teleosts and tetrapods [33]–[35]. Nevertheless, at least some zebrafish pigment mutants have pleiotropic defects, including *panther* (*colony stimulating factor 1 receptor*, *csf1r*) mutants that lack some adult melanophores, all xanthophores, and have defects in osteoclastogenesis and macrophage development, as well as *piccasso* (*erbb3*) mutants that have defects in NCSC-derived adult melanophores and neural crest-derived glia [12],[22],[36].

**Figure 1:**
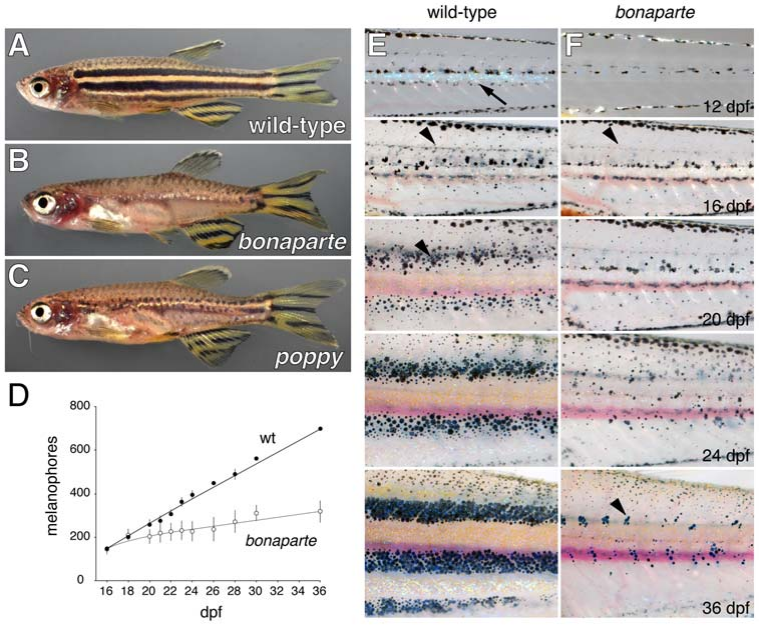
*bonaparte* and *poppy* mutants have fewer metamorphic melanophores, xanthophores, and iridophores. (A) Wild-type adult zebrafish. (B) *bonaparte* mutants lack melanophore and xanthophore stripes on the body but retain stripes in the caudal and anal fins and exhibit a normal pattern of scale melanophores. A few residual iridophores are evident near the horizontal myoseptum. Internal viscera are partially visible owing to the absence of melanophores and iridophores. (C) *poppy* mutants retain partial stripes of melanophores on the body as well as the fins. (D) Time course illustrating the increasingly severe deficit of melanophores (means±SE) in *bonaparte* homozygotes as compared to phenotypically wild-type *bonaparte*/+ siblings followed daily between 16 and 36 days post-fertilization (dpf; *n* = 1 to 3 fish for each genotype across all days). (E,F) Images of individual wild-type (*bonaparte*/+) and *bonaparte* larvae from the onset of pigment pattern metamorphosis (here, 12 dpf) through completion of the adult pigment pattern (36 dpf). *bonaparte* mutants lack early appearing metamorphic iridophores (arrow in E at 12 dpf), but develop dispersed metamorphic melanophores (arrowheads in (E) and (F) at 16 dpf). Subsequently, *bonaparte* mutants do not develop nascent adult stripes as in wild-type (arrowhead in (E) at 20 dpf) and exhibit only residual clusters of melanophores on the flank (arrowhead in (F) at 36 dpf) when wild-type fish exhibit fully formed adult stripes.

In this study, we examine the *bonaparte* mutant, one of a class of mutants that exhibit disrupted adult pigment patterns as well as severe pleiotropic defects in other systems. *bonaparte* mutants lack body stripes yet retain stripes in the fins (Figure 1A and 1B); they are smaller than the wild-type and females are infertile. We find that *bonaparte* mutants have fewer melanophores, xanthophores, and iridophores during adult pigment pattern formation and females have an excess of somatic ovarian tissue. We show that *bonaparte* corresponds to *basonuclin-2* (*bnc2*), a highly conserved locus encoding a nuclear zinc finger protein thought to function in RNA processing [37],[38]. While *bnc2* and the related gene *bnc1* are expressed in diverse and partially overlapping domains, including skin, central nervous system, and gonads, we show with genetic mosaic and image analyses that *bnc2* functions in cells at the surface of the myotome to promote chromatophore persistence. Finally, we demonstrate that *bnc2* is required by each of two genetically distinct populations of melanophores constituting adult stripes, but is not required by chromatophores in the fin, unless *kit* and *csf1r* activity are absent. Our study provides new insights into the development and maintenance of the adult pigment pattern, and identifies an animal model for studying *bnc2* functions in the development of stem cell-derived and other lineages.

## Results

### *bonaparte* mutants have defects in development of metamorphic chromatophores and are female-infertile

We identified the recessive *utr16e1* allele in a forward genetic, early pressure screen for mutations affecting the adult pigment pattern. In comparison to the wild-type, *utr16e1* homozygous adults lacked stripes on the body, except for a few residual chromatophores near the horizontal myoseptum. Chromatophores comprising body stripes are found between the skin and the myotome in a region termed the “hypodermis” [21], [39]–[41], and *bonaparte* mutants had severely reduced numbers of hypodermal melanophores, xanthophores and iridophores. By contrast, stripes in the caudal and anal fin appeared to be normal (Figure 1A and 1B), and *bonaparte* mutants regenerate apparently normal fin pigment patterns after fin amputation (data not shown). Likewise, the pattern of chromatophores covering the scales as well as melanophores in the epidermis were indistinguishable from the wild-type. Besides the pigment pattern defect, mutants were runted and had a patch of medal-like iridophores ventroanteriorly, inspiring the designation *bonaparte^utr16e1^* (*bnp*). Complementation tests and analyses of backcross progeny showed that *bonaparte* is allelic to another mutant, *poppy^j10e1^*, having a somewhat milder pigment pattern phenotype (Figure 1C). We used only the severe allele, *bonaparte^utr16e1^*, in cellular and genetic analyses below.

To determine when the *bonaparte* pigment pattern defect arises, we imaged individual fish through adult pigment pattern formation. Zebrafish express very different pigment patterns during different life cycle phases [1],[20],[42],[43]. In the embryo, a series of melanophore stripes develop at the edges of the myotomes, each containing a few iridophores, whereas xanthophores are widely scattered over the flank. This embryonic/early larval pigment pattern persists until pigment pattern metamorphosis, when new metamorphic melanophores arise scattered over the myotomes and iridophores and xanthophores differentiate just ventral to the horizontal myoseptum. Some of the metamorphic melanophores then migrate to sites of adult stripe formation, whereas additional metamorphic melanophores differentiate already within these stripes, bounding the interstripe of iridophores and xanthophores.

In time-course image series, *bonaparte* mutants and wild-type siblings had melanophore numbers and patterns that were indistinguishable from one another during the embryonic and early larval period. During later development, however, *bonaparte* mutants exhibited increasingly severe melanophore deficiencies (Figure 1D–1F). Similar defects were observed for iridophores and xanthophores. Development of wild-type (*bonaparte*/+) and *bonaparte* mutant phenotypes can be visualized in the Video S1.

**Figure 2:**
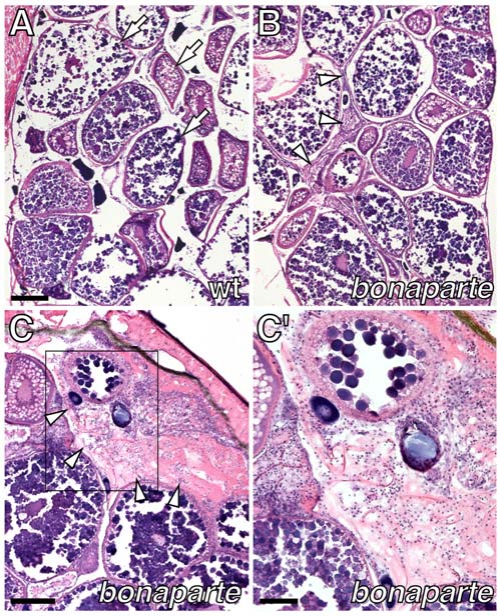
Female infertility and ovarian dysmorphology in *bonaparte* mutants. (A) Cross-section through adult wild-type ovary stained with hematoxylin and eosin, showing oocytes at various stages of development (arrows). (B) Corresponding region of *bonaparte* ovary showing oocytes as well as increased amounts of somatic tissue (arrowheads). (C) Detail of a *bonaparte* ovary showing somatic tissue (arrowheads). (C′) Inset in C. Scale bars: in (A), 200 µm for (A,B); in (C), 200 µm; in (C′), 100 µm.

In addition to pigment pattern and growth defects, *bonaparte* and *poppy* mutant females are infertile. Examination of adult ovaries revealed oocytes at all stages of maturation, but an excess of somatic tissue (Figure 2). Although females do not breed and it was not possible to strip viable eggs for in vitro fertilization, oocytes dissected directly from ovaries were fertilizable at low frequency and the resulting embryos developed normally (data not shown).

### *bonaparte* acts non-autonomously to melanophores during pigment pattern metamorphosis

**Figure 3:**
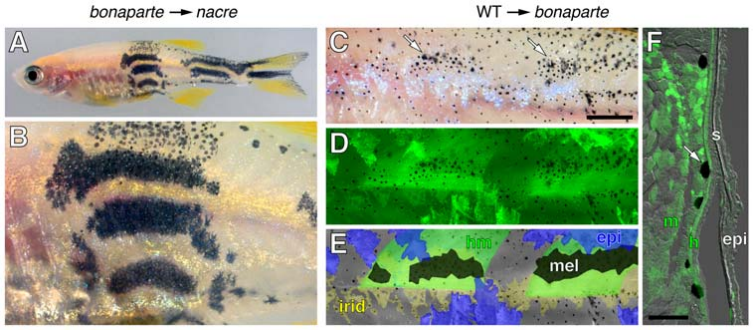
*bonaparte* is required non-autonomously to melanophores. (A,B) *bonaparte* melanophores consistently formed wild-type stripes in melanophore-free *nacre* (*bonaparte* ^+^) hosts (*n* = 8). Shown are low magnification (A) and higher magnification (B) views of the same individual. (C–F) *bonaparte* mutant melanophores consistently formed larger and denser spots when adjacent to wild-type (GFP*^+^*) myotome and hypodermal cells. Shown is a single individual. Melanophores were more spread and patches were larger (arrows in C) and such patches were found above wild-type (GFP*^+^*) muscle (*n* = 3) (D). In this individual, no wild-type melanophores developed, but muscle and epidermis occurred in both overlapping and non-overlapping regions and patches of *bonaparte* mutant melanophores corresponded more closely to the location of muscle than of epidermis. Distributions are shown schematically in E; hm, hypodermal cells and muscle; epi, epidermis; mel, melanophores; irid, iridophores. Melanophore patches were identified by the greater density and more spread morphology of melanophores in fish prior to epinephrine treatment, which causes melanosome translocation towards cell bodies facilitating GFP detection in melanophores and other tissues. The individual shown has not yet fully contracted melanosomes in all melanophores. (F) In transverse section, melanophores (arrow) were adjacent to GFP^+^ muscle (m) as well as a thin layer of hypodermal cells (h), that cannot be seen in whole mount. Scale bars: in (C), 100 µm for (C–E); in F, 100 µm.

We used genetic mosaic analyses to determine whether the gene affected in *bonaparte* acts autonomously or non-autonomously to chromatophore lineages during pigment pattern metamorphosis. We first transplanted cells between blastula stage embryos of *bonaparte* mutant donors and *nacre* mutant hosts [12],[42]. The latter fish have a mutation in *mitfa*, which acts autonomously in melanophore specification [31]. As *nacre* mutants completely lack body melanophores, any melanophores that develop in chimeric individuals are donor-derived. If *bonaparte* acts autonomously to the melanophore lineage, then relatively few melanophores should develop and a defective stripe pattern should form as in *bonaparte* mutants. Conversely, if *bonaparte* acts non-autonomously, then melanophore patterning should be rescued in the *nacre* ^−^ *bonaparte^+^* host background. Since donor embryos were derived from *bonaparte*/+ backcrosses, we genotyped each donor embryo for a linked marker by PCR and reared each chimera individually through development of the adult pigment pattern. In chimeras derived from homozygous mutant *bonaparte* donors, we frequently found large numbers of donor melanophores that persisted into the adult to form wild-type stripes (Figure 3A and 3B) suggesting a function that is non-autonomous to the melanophore lineage.

To ascertain which tissue promotes chromatophore patterning and survival, we next transplanted blastula cells from wild-type donors to *bonaparte* mutant hosts, and we used donors carrying a widely expressed β-actin transgene to distinguish donor cells from host cells. We expected that melanophore patterning should be rescued adjacent to tissues in which *bonaparte* exerts its effects. Although β-actin::GFP is expressed and readily detectable in metamorphic melanophores [12],[42],[44], we found only rare wild-type (GFP+) donor melanophores in *bonaparte* mutant hosts and these were not found in stripes, consistent with a non-autonomous effect of *bonaparte*. By contrast, we observed denser patches of melanophores near other wild-type (GFP+) donor tissues (Figure 3C–3E). When viewed in whole-mount, these regions corresponded more closely to wild-type muscle than to wild-type epidermis. In sections immunohistochemically processed to reveal GFP, however, we always found a layer of wild-type (GFP+) hypodermal cells—superficial to the myotome but beneath the epidermis—adjacent to the dense melanophore patches (Figure 3F). Together, these data indicate that *bonaparte* acts non-autonomously to melanophores—and potentially other chromatophore lineages—during pigment pattern metamorphosis, and this site of action appears to correspond to a layer of cells between myotomes and epidermis.

### *bonaparte* encodes an orthologue of *basonuclin 2*

**Figure 4:**
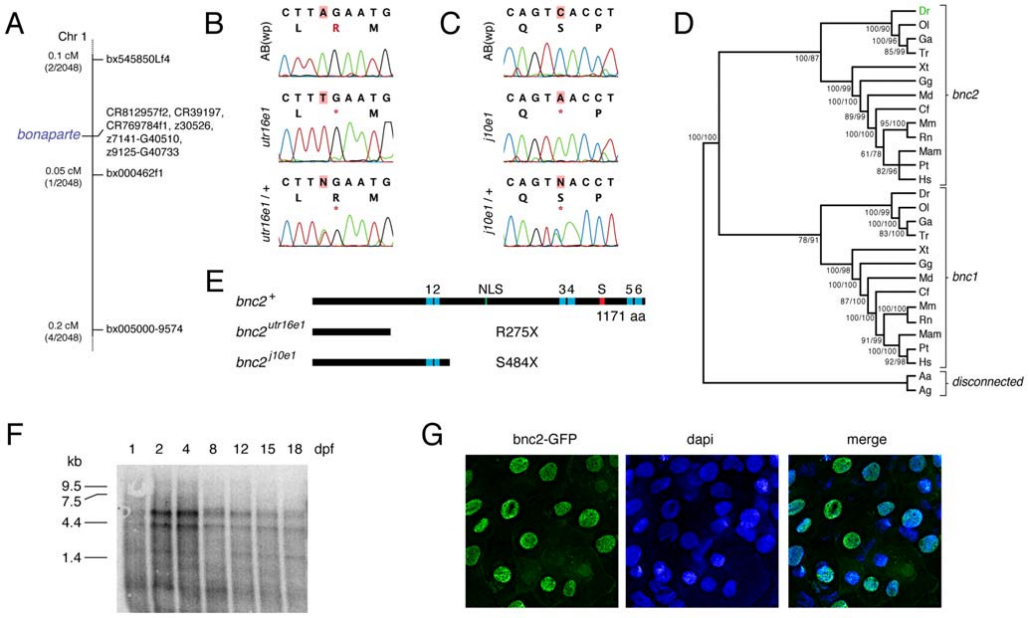
*bonaparte* and *poppy* are mutant alleles of *basonuclin-2* (*bnc2*). (A) Mapping of *bonaparte* to chromosome 1. (B,C) Identification of premature stop codons in *bnc2* alleles of *bonaparte* (B) and *poppy* (C). Shown are wild-type AB^wp^ alleles, homozygous mutant alleles, and heterozygous mutant alleles. *, stop codon. (D) Phylogenetic analysis places zebrafish *bnc2* within the clade of other vertebrate *bnc2* loci. Values in at nodes are bootstrap support based on 1,000 replicates (PAUP heuristic parsimony analysis) followed by posterior probabilities from Bayesian analysis. Abbreviations: Dr, *Danio rerio*; Ol, *Oryzias latipes*; Ga, *Gasterosteus aculeatus*; Tr, *Takifugu rubripes*; Xt, *Xenopus tropicalis*; Gg, *Gallus gallus*; Md, *Monodelphis somestrica*; Cf, *Canis familiaris*; Mm, *Mus musculus*; Rn, *Rattus norvegicus*; Mam, *Macaca mulatta*; Pt; *Pan troglodytes*; Hs, *Homo sapiens*; Aa, *Aedes aegypti*; Ag, *Anopheles gambiae*. (E) Domain structure of bnc2 and predicted truncated proteins. (F) Northern blot of poly(A)+ mRNA extracted from whole fish at the times indicated and probed with a full length *bnc2* RNA probe. Two major *bnc2* isoforms were detected. Equal loading verified by Nanodrop spectrophotometry and β-actin control probe (not shown). (G) bnc2-GFP fusion protein shows nuclear localization in embryo epidermis at 24 hours post-fertilization.

To identify the gene affected in *bonaparte*, we mapped the mutant to an interval on chromosome 1 containing a predicted gene, LOC568199, similar to *basonuclin 2* (*bnc2*) (Figure 4A). Sequencing cDNAs for this locus from *utr16e1* and *j10e1* revealed premature stop codons in both genetic backgrounds (Figure 4B and 4C) and the *utr16e1* lesion segregated with the *bonaparte* mutant phenotype (data not shown). We cloned a presumptive full-length open reading frame (GenBank accession number GQ229411) and, using phylogenetic reconstruction, we found this locus to be orthologous to *bnc2* (Figure 4D). Computational searches of zebrafish genome sequence and expressed sequence tags revealed only a single *bnc2* locus.

bnc2 has conserved domains comprising three pairs of zinc fingers, a nuclear localization signal, and a serine stripe (Figure 4E) [38],[45]. The predicted protein encoded by *utr16e1* lacks all of these domains whereas that encoded by *j10e1* lacks all but the first pair of zinc fingers. Whereas *utr16e1* is likely to be a null allele, we cannot exclude the possibility of residual activity, as might result from translational read-through of premature termination codons, or, particularly for *j10e1*, from residual activity of a truncated protein [46]. Quantitative PCR revealed only marginally reduced transcript levels in *bonaparte* mutants (expression ratios of *bonaparte* to wild-type: 0.50–0.76).

Mammalian *bnc2* orthologues exhibit a variety of splice variants, and have the potential to generate many additional isoforms [45],[46]. We asked if zebrafish *bnc2* is expressed in multiple isoforms as well. In Northern blots of mRNA isolated from whole fish at a range of ages, we detected large transcripts of ∼4 and ∼5 kb as well as a minor transcript ∼1.8 kb, suggesting the presence of alternative splicing products (Figure 4F).

In mammals, bnc2 localizes constitutively to the nucleus, whereas the closely related bnc1 shuttles between nucleus and cytoplasm depending on proliferation state. These different behaviors are thought to result from an amino acid substitution at position 537 (bnc2, pro; bnc1, ser) of the nuclear localization signal [37],[45],[47]. Zebrafish bnc2 exhibits the bnc2-typical proline residue at this site, and we found that a bnc2-EGFP fusion protein localized to the nucleus in 24 hours post-fertilization embryos injected with mRNA at the one-cell stage (Figure 4G).

### *bnc2* is expressed in diverse tissues including the skin during pigment pattern metamorphosis and exhibits partially overlapping expression domains with *bnc1*

**Figure 5:**
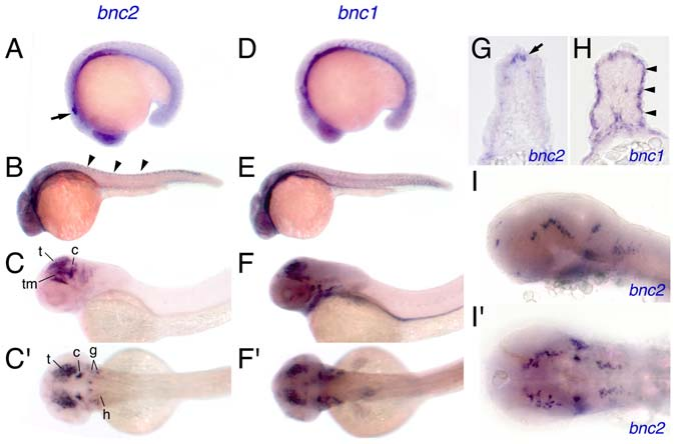
Embryonic expression of *bnc2* and *bnc1*. (A) At 18 hpf, *bnc2* was expressed broadly in ectodermal and mesodermal tissues with particular expression visible in otocyst (arrow). (B) At 24 hpf, *bnc2* transcript was present within the dorsal neural tube (arrowheads) and within the anterior central nervous system. (C,C′) At 48 hpf, strong *bnc2* expression was limited to the brain and cranial ganglia shown in lateral (C) and dorsal (C′) aspects. t, tectum; tm, tegmentum; c, cerebellum; g, cranial ganglia; h, hindbrain neurons. (D) *bnc1* was expressed similarly to *bnc2* at 18 hpf. (E) At 24 hpf, *bnc1* transcripts were seen in the myotomes and throughout the head anteriorly. (F,F′) At 48 hpf, *bnc1* was expressed widely at low levels and at higher levels within the brain, cranial ganglia, and pronephric duct. (G) In transverse section at 24 hpf, *bnc2* transcript was present in the dorsal neural tube (arrow). (H) Simultaneously, *bnc1* transcript could be seen lining the outer edges of the myotome (arrowheads). (I,I′) Details of the *bnc2* expression in the brain and cranial ganglia at 36 hpf, from laterally (I) and dorsally (I′).

We examined the temporal and spatial domains of *bnc2* expression. By 18 hours post fertilization (hpf), we observed low levels of expression in diverse tissues including both ectoderm and mesoderm. Higher levels of expression were present in the eye, otic vesicle, and cells within the dorsal neural tube (Figure 5A, 5B, and 5G). In later embryos, high levels of *bnc2* transcript were limited to the brain as well as cranial ganglia (Figure 5C and 5I).

**Figure 6:**
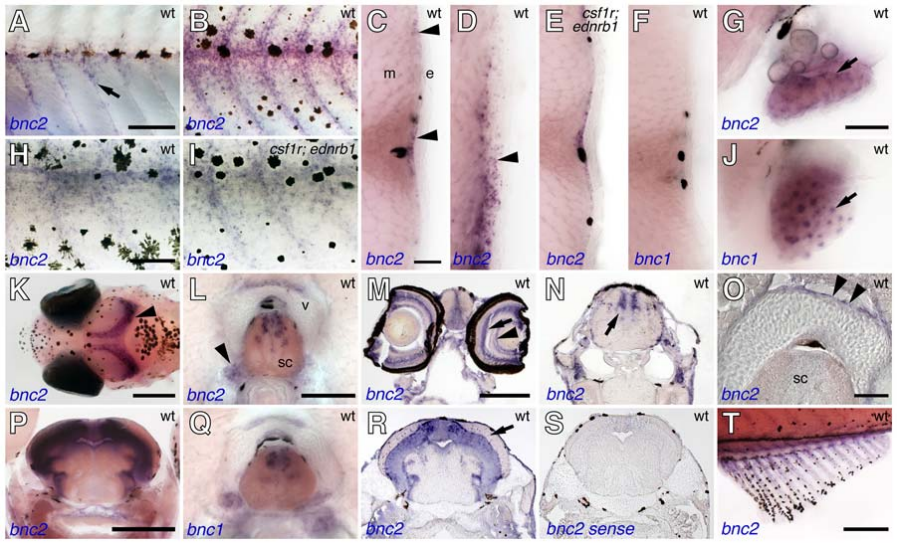
*bnc2* and *bnc1* expression in diverse tissues during the larval-to-adult transformation. (A) *bnc2* transcript was seen in hypodermal cells during early stages of pigment pattern metamorphosis (7.0 mm standardized standard length; 7 SSL [79]). (B) *bnc2+* cells were more widely scattered over the flank during middle stages of pigment pattern metamorphosis (∼10 mm SL). (C) In cross sections, *bnc2+* cells (purple stained, arrowheads) occurred at the level of the hypodermis, near the horizontal myoseptum as well as further dorsally and ventrally (∼9 mm SL). Black melanophores also were present in the epidermis (e) and myotome (m). (D) In further ventral regions at the same stage, *bnc2+* cells were present both within the hypodermis and in the epidermis (arrowhead). (E) The distribution of *bnc2*+ cells in *csf1r; ednrb1* double mutants was similar to that of wild-type, suggesting *bnc2+* cells are not xanthophores or iridophores. (F) In contrast, *bnc1* was not expressed in the skin or hypodermal region at comparable stages as detectable by in situ hybridization. (G) In the ovary, *bnc2* was expressed by somatic cells (arrow). (H,I) Higher magnification views of *bnc2* expression at 10 SSL in wild-type (H) and *csf1r; ednrb1* double mutants (I), with similar staining patterns. (J) In contrast to *bnc2*, *bnc1* transcript was seen in ovarian oocytes. (K) Dorsal head of 9 SSL larva showing *bnc2* expression in the brain (arrowhead). (L) *bnc2+* cells in dorsal spinal cord (sc), ganglia (arrowhead), and dorsal superficial cells of vertebral centrum (v). (M) Transverse cryosection through the head revealed expression in the eye, including the inner nuclear layer and ganglion cell layer (arrow and arrowhead, respectively) and in the diencephalon. (N) Cryosection showing *bnc2* staining in the hindbrain (arrow). (O) Detail showing staining of superficial cells of centrum (arrowheads). (P) Transverse vibratome section through midbrain showing *bnc2+* cells in periventricular grey zone (PGZ) of the tectum. (Q) Staining for *bnc1* in dorsal spinal cord. (R). Cryosection through midbrain showing broad staining within the PGZ as well as individual scattered *bnc2+* cells within the tectum (arrow). (S) *bnc2* sense control probe. (T) bnc2 was also expressed in the fins, including the anal fin shown here (∼12 SSL). Scale bars: in (A), 100 µm for (A,B); in (H), 50 µm for (H,I); in (C), 20 µm for (C–F); in (G), 40 µm for (G,J); in (K), 200 µm; in (L), 50 *µ*m, for (L,Q); in (M), 200 µm for (M,N,R,S); in (O), 20 µm; in (P), 200 µm; in (T), 200 µm.

During the larval-to-adult transformation, *bnc2* was expressed by cells between the myotome and epidermis, initially in regions near the horizontal myoseptum and extending dorsally and ventrally near the vertical myosepta (Figure 6A). During later development, bnc2+ cells were more widely distributed but still were found at greatest densities in the vicinity of the myosepta (Figure 6B). In cross sections, these cells were immediately superficial to the myotome, but also could be found within the epidermis, particularly ventrally (Figure 6C and 6D). We also observed *bnc2+* somatic cells within the ovaries (Figure 6G), brain, dorsal spinal cord, and eye, as well as in superficial cells covering the vertebrae (Figure 6K–6P and 6R). Moreover, we found abundant bnc2 expression in the fins, despite their apparently normal pigment patterns (Figure 6T). Finally, we also detected abundant *bnc2* expression by RT-PCR in adult gut as well as kidney and testes (data not shown).

Since the above genetic mosaic analyses only addressed autonomy relative to the melanophore lineage, we considered the possibility that *bnc2+* cells between myotome and epidermis might be xanthophores or iridophores and that bnc2 effects on melanophores might occur through these lineages. This was an appealing idea as interactions between melanophores and xanthophores are necessary for stripe formation, and failures in such interactions produce pigment pattern defects reminiscent of that seen in *bonaparte* mutants [24],[42],[48]. To test this possibility, we examined the distribution of *bnc2+* cells in fish doubly mutant for *colony stimulating factor-1 receptor* (*csf1r*) and *endothelin receptor b1* (*ednrb1*), which lack xanthophores and iridophores while retaining melanophores [22]. We could not detect differences in *bnc2* expression between wild-type larvae and *csf1r; ednrb1* mutants (Figure 6E and 6I). Furthermore, the distributions of cells expressing molecular markers of xanthophores, iridophores, and their precursors did not correspond to the distributions of *bnc2+* cells in wild-type fish (see below). Together these findings indicate that *bnc2+* cells in the hypodermis are not chromatophores or their precursors.

In mouse and human adults, *bnc2* is expressed in a variety of tissues including skin, gut, liver, ovary, testis, and kidney; in the mouse embryo, a *bnc2*-driven lacZ transgene is expressed in the gut, the craniofacial skeleton, and the joints of the limbs [38],[45],[49]. By contrast, the closely related gene, *bnc1*, has a more restricted domain of expression in adult mammals, limited primarily to skin and testes and its embryonic expression has not been reported [37],[50],[51]. We asked whether, *bnc1* exhibits expression domains in zebrafish similar to those of mammals. In embryos, we found relatively widespread *bnc1* expression, including the somites and presumptive slow muscle fibers of the lateral myotome, the pronephric duct, and in partially overlapping domains with *bnc2* in the brain and eye (Figure 5D–5F and 5H). During the larval-to-adult transformation, we observed *bnc1* expression in brain and spinal cord and also in oocytes (Figure 6J and 6Q). Though we did not detect *bnc1* transcript in the skin by in situ hybridization during the larval-to-adult transformation (Figure 6F), we did find *bnc1* expression by RT-PCR in adult skin as well as in kidney and testes (data not shown). Thus, *bnc1* and *bnc2* have partially overlapping as well as unique expression domains.

### Chromatophore differentiation followed by death in *bonaparte* mutants

**Figure 7:**
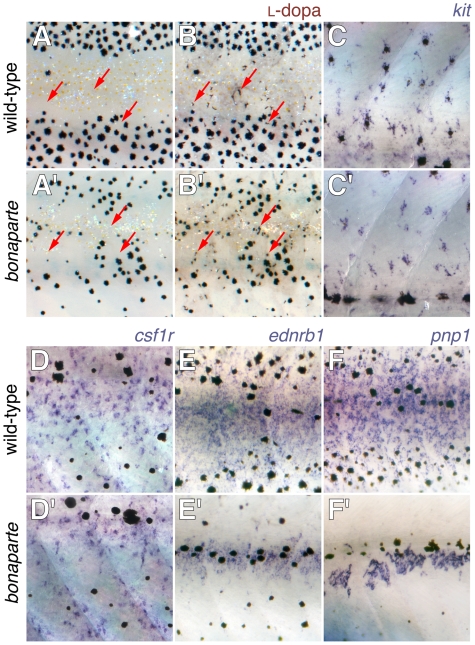
Pigment cell specification and early differentiation in *bonaparte (bnc2)* mutants. Shown are corresponding views from the flanks of mid-metamorphic 8–10 SSL larvae with wild-type above and *bonaparte* (*bnc2*) mutants below. (A,B) Staining with L-dopa revealed previously cryptic cells that are competent to synthesize melanin (arrows) in both wild-type and mutant. (A,A′) Prior to staining. (B,B′) After staining. (C,C′) *kit+* melanoblasts were detectable in both wild-type and bonaparte mutants. (D,D′) *csf1r+* xanthophore precursors were found in both backgrounds. (E,E′) *ednrb1+* cells that may be iridophores as well as other chromatophore precursors are present though fewer in *bonaparte*. (F,F′) *purine nucleoside phosphorylase 1* (*pnp1*) expressing iridophore precursors (DMP unpublished data) are present though dramatically reduced in number in *bonaparte*.

To better understand how *bnc2* promotes adult pigment pattern formation, we examined chromatophore development during metamorphosis. The reduced complement of chromatophores in *bonaparte* mutants could indicate defects in specification and differentiation. To test this possibility, we examined the distributions of cells expressing early markers of these lineages [22],[28],[29],[43]. Melanophore precursors detected by L-dopa staining or *kit* expression were similarly numerous between wild-type and *bonaparte* mutants, as were xanthophore precursors, detected by expression of *colony stimulating factor-1 receptor* (*csf1r*) or *aldehyde oxidase 3* (Figure 7A–7D and data not shown). By contrast, precursors to all three chromatophore classes are likely to be marked by *ednrb1* expression and we found fewer cells expressing *ednrb1* in *bonaparte* mutants than in wild-type (Figure 7E). Since differentiated iridophores also express *ednrb1*, the difference between genotypes may reflect the reduced complement of iridophores in *bonaparte* mutants. Consistent with this interpretation, iridophore precursors marked specifically by *purine nucleoside phosphorylase 1* (*pnp1*; DMP unpublished; K. Curran and D. Raible, personal communication) were fewer and their distribution more limited in *bonaparte* (Figure 7F). These data do not indicate gross defects in the specification and early differentiation of melanophores or xanthophores, whereas early iridophore differentiation and dispersal are impaired.

**Figure 8:**
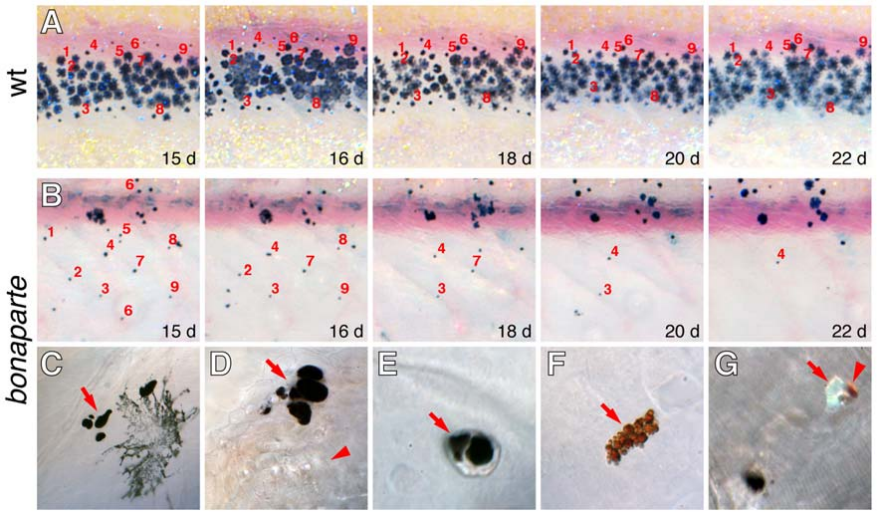
*bnc2* is required for survival of melanophores, xanthophores, and iridophores. Shown are repeated images from single larvae, with specific cells numbered. (A) In wild-type, individual cells could be followed through adult stripe formation. (B) In *bonaparte* mutants, individual cells were lost over a period of several days, corresponding to late stages of pigment pattern metamorphosis (11–14 SSL). (C–G) Higher magnification views showing chromatophore death and extrusion in *bonaparte* mutants. (C) Melanin-containing cellular fragments beneath the skin (arrow), adjacent to an intact melanophore. (D) A cluster of melanin-containing fragments at the surface of the epidermis (arrow), adjacent to a lateral line neuromast (arrowhead). (E) Melanin-containing extrusion body (arrow) that has just been extruded but is still adherent to the outer epidermis. (F) Pteridine and carotenoid-containing cellular debris at the epidermal surface, indicative of xanthophore loss. (G) Extrusion bodies contaiing melanin (lower left) as well as single body containing both xanthophore pigment and iridophore reflecting platelets (arrowhead and arrow, respectively). Typically, at least ten extrusion bodies could be found per individual examined.

To see if reduced chromatophore numbers might result from defects in survival, we examined the persistence of melanophores in image series of individual larvae. In the wild-type, we did not observe the loss of differentiated melanophores, consistent with previous observations [52] (Figure 8A). In *bonaparte* mutants, however, melanophores were frequently lost during pigment pattern metamorphosis (Figure 8B). Closer examination revealed that all three classes of pigment cell are lost by extrusion through the skin, as has been seen in other mutants [28],[42] (Figure 8C–8G). These data implicate *bnc2* in promoting chromatophore survival during pigment pattern metamorphosis.

### *bnc2* requirement by early and late metamorphic melanophores

**Figure 9:**
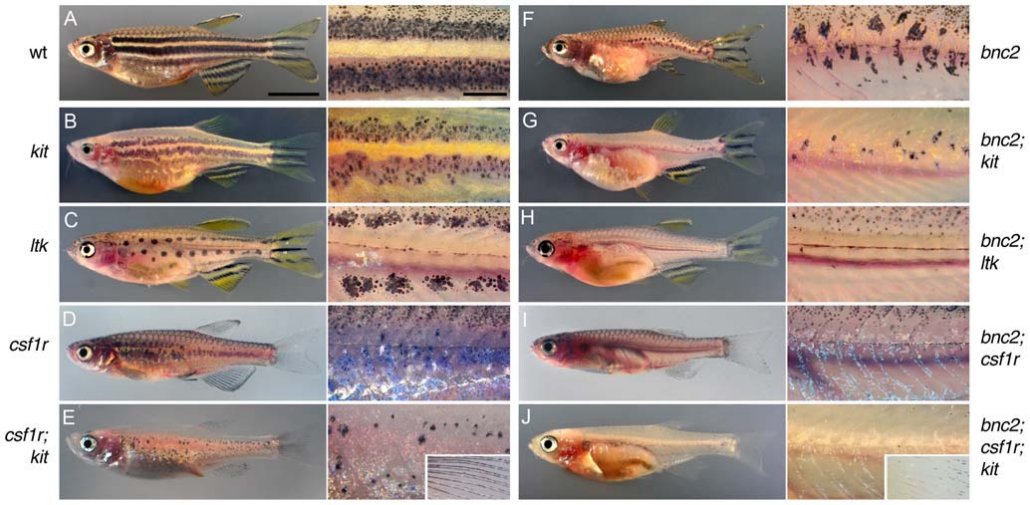
*bnc2* promotes both early and late metamorphic stripe melanophores, but is required by fin melanophores only in the absence of *csf1r* and *kit*. Shown on the left are wild-type (A), the single mutant phenotypes of *kit* (B), *ltk* (C), *csf1r* (D) and the double mutant phenotype of *csf1r; kit* (E). Shown on the right is *bonaparte* (*bnc2*) (F) and the corresponding double and triple mutants with *bonaparte* (G–J). Whole fish images as well as details of the flank are shown. *kit* mutants lack EM melanophores but retain LM melanophores; *kit* mutants and all combinations with *kit* also lack scale melanophores [e.g., arrowhead in (B)]. *ltk* mutants lack both LM melanophores and body iridophores, but retain EM melanophores. *csf1r* mutants lack LM melanophores and all xanthophores, but retain EM melanophores in a dispersed pattern. *csf1r; kit* double mutants lack nearly all hypodermal body melanophores and lack all xanthophores, but retain melanophores in the fins (inset). All double and triple mutant combinations with *bonaparte* exhibited fewer hypodermal melanophores than the corresponding non-compound mutants. Remaining body melanophores are almost entirely found in association with scales (e.g., arrowheads in H′). *bnc2; csf1r; kit* mutants lacked virtually all body chromatophores and exhibited only a few residual melanophores at the tips of the fins (arrow), as well as a “medallion” of iridophores over the cardiac region. Scale in (A), 5 mm for (A–J); scale in (A′), 2 mm for (A′–J′).

The pigment pattern of danios includes distinct populations of hypodermal melanophores: early appearing metamorphic (EM) melanophores and late appearing metamorphic (LM) melanophores [20], [22], [53]–[55]. EM melanophores require the kit receptor tyrosine kinase. By contrast, LM melanophores require leukocyte tyrosine kinase (ltk) and csf1r, but do not require kit (Figure 9). The temporally increasing severity of the melanophore deficiency in *bonaparte* mutants suggested that *bnc2* might be required by LM, but not EM, melanophores. If so, fish doubly mutant for *bnc2* and *kit* (which ablates EM melanophores) should lack virtually all melanophores. Consistent with LM melanophores requiring *bnc2*, we found that *bnc2; kit* double mutants lacked nearly all hypodermal melanophores (Figure 9G). However, contrary to the above prediction, double mutant phenotypes also supported a role for *bnc2* in promoting EM melanophore development, as both *bnc2; ltk* and *bnc2; csf1r* mutants lacked nearly all hypodermal melanophores as well (Figure 9H and 9I). These data indicate that *bnc2* is required both by EM and LM melanophores.

Previous analyses also have indicated different genetic requirements for scale and fin melanophores as compared to hypodermal melanophores [20],[56]. Scale melanophores are absent in *kit* mutants, but we did not uncover additional defects in *bnc2; kit* double mutants (Figure 9G). Fin melanophores were found in all single and double mutants (Figure 9B–9I), but were mostly absent in *bonaparte; kit; csf1r* triple mutants (Figure 9J). Thus, *bnc2* is not essential for scale melanophores, but is required by fin melanophores in the absence of *csf1r* and *kit*.

## Discussion

Our study identifies *bnc2* as a critical factor in adult pigment pattern development. We found that *bnc2* acts non-autonomously to metamorphic melanophores and is expressed by hypodermal cells in contact with melanophores, xanthophores, and iridophores. In *bonaparte* (*bnc2*) mutants, all three chromatophores differentiated but ultimately were lost, resulting in gross defects in stripe formation. *bnc2* was expressed in other tissues as well, including somatic cells of the ovaries, and *bonaparte* (*bnc2*) mutant females were both infertile and exhibited excess somatic tissue within the ovaries. These findings reveal a novel gene required for chromatophore patterning and survival and establish an animal model in which to further dissect roles for *bnc2* in development and homeostasis.

bnc2 was first identified in avian and mammalian systems by similarity to bnc1 [38],[45],[57]. Both proteins contain three pairs of zinc fingers as well as nuclear localization signals and serine-rich regions. As compared to other zinc finger proteins, bnc2 exhibits very high conservation among species whereas bnc1 exhibits a below-average degree of conservation [37]. In mammals, *bnc2* is expressed in a wide range of adult tissues including ovary, testes, skin, gut, lung, and kidney, and bnc2 protein is found constitutively within the nucleus, where it localizes to nuclear speckles, suggesting a role in RNA processing [37]. We found that a zebrafish bnc2:GFP fusion protein translocates to the nucleus as well, though whether or not it localizes within nuclear speckles will require further investigation. Zebrafish *bnc2* was expressed more widely than has been reported in mammals, with particularly abundant expression in the brain and spinal cord. A recently reported mouse mutant for *bnc2* dies neonatally and exhibits cleft pallete and other craniofacial abnormalities, owing to defects in the proliferation of *bnc2*-expressing mesenchymal cells [49]. Further exploration of evolutionary changes in *bnc2* expression and function should be especially interesting.

In contrast to *bnc2*, mammalian *bnc1* is expressed in a more limited domain in adults, including oocytes, spermatogonia, epidermis, and corneal epithelium [38],[45],[58],[59] and bnc1 protein shuttles between nucleus and cytoplasm depending on cellular proliferative state [37],[47],[60]. bnc1 binds to ribosomal RNA gene (rDNA) promoters and possibly other targets, and mouse null mutants for this locus exhibit relatively subtle phenotypes, limited to defects in corneal homeostasis and wound repair [61]–[64]. Female mice transgenic for an oocyte-targeted *bnc1* interfering RNA transgene exhibit defective oocyte morphology, reduced rates of transcription, and reduced fertility [58]. Our finding that *bnc1* transcript localizes to oocytes, whereas *bnc2* transcript localizes to somatic cells in the ovaries, suggests potential roles for both genes in oogenesis and reproduction. The expression of both genes within the central nervous system suggests additional roles in neurogenesis or neural maintenance deserving of further exploration.

Our finding that *bonaparte* corresponds to *bnc2* is not the first time *bnc2* has been implicated in a pigment mutant phenotype. In mouse, the *white-based brown* (*Tyrp1^B–w^*) allele causes the absence of melanin at the base of the hair shaft, owing to melanocyte death late in the hair cycle [65],[66]. The molecular basis for this phenotype lies in a chromosomal inversion that brings the nearby Bnc2 locus adjacent to *Tyrp1*, which encodes the melanin-synthesis enzyme, tyrosinase-related protein 1 [67]. This rearrangement results in the production of a fusion protein consisting of Bnc2 and the first exon of Tyrp1 that is expressed in melanocytes and other sites including the eye, skin and kidney. Death of melanocytes in *white-based brown* mice is thought to result from the autonomous overexpression of Bnc2 within these cells.

In contrast to *white-based brown*, our analyses of *bonaparte* mutants are the first to demonstrate a role for the native bnc2 gene in pigment cell development and patterning. Cell transplantation, gene expression analyses, and time-course imaging together indicated that *bnc2* acts non-autonomously to chromatophore lineages to promote chromatophore persistence in the hypodermis: melanophores, xanthophores, and iridophores all differentiated but then could be found as cellular fragments, within extrusion bodies in the skin, or both. This phenotype is similar to zebrafish *kit* mutants, in which embryonic/early larval melanophores die and are lost by extrusion, conditional alleles of zebrafish *csf1r* mutants, in which melanophores and xanthophores die and are extruded following csf1r inactivation, and the naturally occurring phenotype of *D. albolineatus*, in which melanophores are extruded during pigment pattern metamorphosis [28],[43],[48]. Extrusion of chromatophores through the skin in these and other fishes [68] is somewhat reminiscent of epithelial clone extrusion in *Drosophila* wing disks deficient for Decapentaplegic [69], though the precise mechanisms remain unclear in either instance. Given the phylogenetic and developmental differences between teleosts and *Drosophila*, this mode of cellular loss across epithelia may be more widespread across taxa as well as organ systems. In addition to chromatophore loss, we observed fewer iridophore precursors, suggesting a defect affecting the differentiation of this lineage. Likewise, we cannot exclude the possibility that pigment pattern defects in *bonaparte* arise in part due to subtle defects in melanophore or xanthophore specification and differentiation, or the loss of chromatoblasts prior to their differentiation.

The precise identity and function of the *bnc2*-expressing cells affecting chromatophores remains unclear. Whereas *bnc2* in mammals is expressed by keratinocytes [45], zebrafish *bnc2* was expressed in cells within the hypodermis at the surface of the myotome, and within the epidermis itself. The distribution of these cells was distinct from that of cells expressing markers of chromatophore lineages, as well as presumptive markers of dermis or epidermis (e.g., *keratin4*, *col1a2*, *dermo1/twist2*; data not shown). Moreover, *bonaparte* (*bnc2*) mutants did not exhibit gross defects in the expression domains of such dermal or epidermal markers, the histological appearance of the skin, rates of skin cell proliferation as assayed by BrdU or EdU incorporation, or rates of skin cell death as assayed by acridine orange staining (data not shown). Nevertheless our analyses to date cannot rule out very subtle or developmentally transient defects in dermal or epidermal lineages.

Given its non-autonomous effects, we speculate that *bnc2* may be required for producing or localizing a survival factor for chromatophores. Most candidate factors identified to date would not be expected to affect all three chromatophore classes, raising the possibilities of a previously unidentified factor, or that loss of one or two chromatophore classes ultimately leads to the loss of all three classes. Consistent with this idea, interactions promoting survival and migration occur between melanophores and xanthophores, and additional interactions might involve iridophores [21],[23],[24],[48],[70]. It will be interesting to discover if chromatophore loss and female infertility are linked through a common *bnc2*-dependent survival factor, as in *Kit* mutant mice [17].

A striking feature of the *bonaparte* mutant phenotype was the presence of seemingly normal chromatophores and patterns covering the scales and in the fins, despite the marked disruption of hypodermal chromatophores. Indeed, the chromatophore phenotype did not correlate perfectly with *bnc2* expression, as we found abundant *bnc2* transcript in the fins. Since fin pigment patterns readily regenerate [56],[71], a conceivable explanation would be that chromatophores are continually lost from the fins but rapidly replenished, producing a normal pattern despite an abnormally high cellular turnover. Nevertheless, analyses using the melanization inhibitor phenylthiourea to examine rates of turnover failed to reveal differences between the wild-type and *bonaparte* mutants (ML and DP unpublished). A second possible explanation would lie with the arrangements of chromatophores themselves, if, for instance, chromatophore stripes in the hypodermis differ fundamentally from chromatophore stripes in the fins. Nevertheless, ultrastructural analyses suggest broad similarities in chromatophore arrangements within stripes of both regions [39]. That fin chromatophores did require *bnc2* in the absence of *kit* and *csf1r* activity suggests a role for a still unidentified survival factor present in this tissue but not present in the hypodermis at corresponding stages; that fin chromatophores were lost in *bnc2; csf1r; kit* triple mutants, but not *bnc2; csf1r* or *bnc2; kit* double mutants further indicates that csf1r and kit signals are at least partially redundant in these cells.

Our study adds to the growing evidence that different populations of chromatophores, as well as mammalian melanocytes, exhibit very different genetic requirements. Such populations may be temporally separated, as in *bonaparte* (*bnc2*), *rose* (*ednrb1*) and *picasso* (*erbb3*) mutants, which all exhibit embryonic/early larval pigment patterns indistinguishable from the wild-type while displaying profound adult pattern defects (this study; [12],[20],[29],[72],[73]). Such differences may reflect differential requirements of cells derived directly from neural crest cells and those derived from latent NCSCs during adult pattern formation or regeneration. Genetically distinct populations also may be spatially separated, as for hypodermal vs. fin and scale chromatophores (this study; [20]) or non-cutaneous and cutaneous melanocytes in mouse [74]. The dissociability of pigment cell populations is likely to confer evolutionary flexibility in the sorts of patterns that can arise and be selected in nature. The existence of different cell populations with different genetic requirements also has biomedical implications, as pigment disorders and melanomas arising from different populations may respond differentially to particular therapies [75]–[77].

## Materials and Methods

### Fish rearing, stocks, and genetic mapping

Adult fish were maintained at 28–29°C, 14L:10D. Embryonic staging followed [78] and post-embryonic staging used standardized standard length (SSL) measurements following [79]. The *bonaparte^utr16e1^* and *poppy^j10e1^* alleles were induced by *N*-ethyl-*N*-nitrosourea mutagenesis in AB^wp^ and SJD backgrounds, respectively. Mapping families were generated by crosses of *bonaparte^utr16e1^* to wik, followed by backcrossing to *bonaparte^utr16e1^*, with 2048 backcross progeny used for genetic linkage mapping. The mutant was placed initially on chromosome 1 by bulked segregant analysis, with subsequent fine mapping using published microsatellite markers and single nucleotide polymorphisms. For genotyping *bonaparte^utr16e1^* we used PCR primers that spanned the mutant lesion, which results in the loss of a diagnostic *Dde I* restriction site. Segregation of the mutant phenotype and lesion was confirmed by sequencing of recombinant mapping cross individuals. Other stocks used included: WT(WA), a wild-type stock generated de novo each generation by crossing the inbred lines AB^wp^ and wik, and used for histology, as well as *kit^b5^*, *csf1r^j4blue^*, *ltk^j9s1^*, and *nacre^w2^*. Double and triple mutants were generated by standard intercrosses and genotyped by PCR using primers diagnostic for mutant lesions. All work in this study was conducted in accordance with IACUC regulations through University of Washington animal care protocol 4094-01.

### Histology and northern blotting

In situ hybridization and L-dopa staining followed standard procedures [22],[43]. For some analyses, fish were vibratome-sectioned at 200–250 µm prior to hybridization, whereas other specimens were sectioned by vibratome or cryostat following staining. Both approaches gave similar results. Detailed methods for in situ hybridization of post-embryonic specimens are available at http://protist.biology.washington.edu/dparichy/. Riboprobes used were targeted to *bnc2*, *bnc1*, *kit* [28], *csf1r* (*fms*) [22], *ednrb1* [29], *aldehyde oxidase 3/xanthine dehydrogenase* [22], and *pnp1* (see Results). Probes for *bnc2* used in both Northern blotting and in situ hybridization were transcribed from a full length clone and should recognize all splice forms. The probe for *bnc1* was targeted to a 678 bp region between the middle and 3′ terminus of the transcript. Staining with hematoxylin and eosin as well as Northern blotting followed standard protocols.

### Cell transplantation and microinjection

Chimeric embryos were generated by transplanting cells at blastula or shield stages and resulting embryos reared through pigment pattern metamorphosis. Genotypes used were: *bonaparte^utr16e1^*, *mitfa^w2^*, and wild-type transgenic for GFP driven by a ubiquitously expressed β-actin promoter (generously provided by K. Poss). For analyses using GFP, we avoided potential quenching by melanin in melanophores by treating fish with epinephrine prior to imaging, thereby causing melanosomes to be translocated away from cellular processes and towards cell bodies. Microinjection of one cell stage embryos followed standard methods.

### Imaging and image analyses

Larvae were viewed with Olympus SZX-12 or Zeiss Discovery stereomicroscopes or with a Zeiss Observer inverted microscope. Images were collected using Axiocam HR and MR3 cameras using Axiovision 4.1. For thick specimens, stacks of images were collected and processed using the Zeiss Axiovision Extended Focus or 6D Acquisition modules. For time-course analyses, individual fish from a *bonaparte*/+ backcross were imaged and phenotypes determined retrospectively. These fish were reared individually and imaged daily following brief anesthetization. Images were subsequently aligned and scaled in Adobe Photoshop CS4 to control for growth.

### Phylogenetic analysis

bnc2 and bnc1 amino acid sequences were analyzed in PAUP*4.0 (Sinauer Associates) and MrBayes [80], using the invertebrate Disconnected protein as outgroup. Accession numbers or Ensembl protein identification numbers were in order of appearance in Figure 4D: ACT31324, ENSORLP00000008983, ENSGACP00000022991, ENSTRUP00000025985, ENSXETP00000039119, ENSGALP00000024317, XP_001373237, XP_853948, NP_766458, NP_001100136, XP_001110426, XP_520498, NP_060107, XP_697231, ENSORLP00000023794, ENSGACP00000018944, ENSTRUP00000009908, ENSXETP00000035077, XP_425076, XP_001366113, XP_545874, NP_031588, NP_001102386, XP_001111612, XP_523140, NP_001708, XP_001663557, XP_322062.

## Supporting Information

Video S1 Adult pigment pattern formation in wild-type and *bonaparte* mutants. Shown are wild-type (*bonaparte*/+) and *bonaparte* mutant siblings over 20 days of pigment pattern metamorphosis. New melanophores appear dispersed over the flank in both backgrounds but most of these are lost during later pigment pattern metamorphosis in *bonaparte*. Individual images were re-scaled to control for growth, allowing changes in pigment pattern to be more clearly apparent.
(9.44 MB MOV)
